# Marion Koopmans: greater regional capacity to fight disease outbreaks

**DOI:** 10.2471/BLT.18.030218

**Published:** 2018-02-01

## Abstract

Marion Koopmans tells Fiona Fleck why the world needs a publicly-funded network of hubs in all regions with local experts able to respond to infectious disease threats as they emerge.

Q: What is an emerging infectious disease or pathogen and why are these important for public health today? How did you become interested in researching these diseases?

A: An emerging disease or pathogen is a disease that is new, or has been detected in a new region or with manifestations that differ from what was previously recognized. I’ve been investigating outbreaks for much of my career, but I first experienced the major public health impact of an emerging infectious diseases outbreak in 2003, when we had a large avian influenza (A) H7N7 outbreak in the Netherlands and I was responsible for national laboratory preparedness and response. I realized how fast such events can develop, the difficulty of working with an unknown disease and the challenge of ensuring a coordinated response between veterinary and public health experts. We thought we were prepared, but it was not easy. Since then, I found similar challenges in the responses to the severe acute respiratory syndrome (SARS) outbreak in 2003, a Marburg virus importation in 2008, other avian influenza virus outbreaks when the influenza (A) H5N1 viruses started to spread from China, and in the responses to Ebola virus disease, Middle East Respiratory Syndrome (MERS) and Zika.

Q: How were you involved in these outbreaks?

A: When these outbreaks occurred, laboratories in the Netherlands had to be ready to test suspected cases. During the SARS outbreak, I was involved in risk assessments of the potential for foodborne transmission. Regarding MERS and Zika, I was – and still am – involved in research with colleagues in the affected countries. During the 2014–16 Ebola outbreak, the Netherlands donated three mobile laboratories to Liberia and Sierra Leone, which were deployed and staffed by our medical centre.

“A public health laboratory should be able to detect many diseases with the resources and manpower they currently use for one disease.”

Q: What are the public health challenges posed by emerging infectious diseases?

A: The main challenge is the fragmented way in which we do surveillance and respond to outbreaks of these diseases. We have well-established and reasonably funded networks and laboratories with defined roles and responsibilities for vaccine-preventable diseases, foodborne diseases, some blood-borne diseases and, increasingly, antimicrobial resistance. However, they are not set up for general emerging disease preparedness and response. Our disease–specific response is unsustainable and unnecessary. A public health laboratory should be able to detect many diseases with the resources and manpower currently used for one disease, in collaboration with animal and environmental health experts – this is known as the One Health approach. This generic model should be our goal. My view – and that of many of my colleagues – is that we need a publicly-funded network of expert centres in all regions that work together on a routine basis, not just when there is an outbreak, each working on many emerging infectious diseases.

Q: Doesn’t the Global Outbreak Alert and Response Network (GOARN) do this?

A: GOARN mobilizes experts from its network of institutions and laboratories around the world to respond to outbreaks in other countries. But this mechanism wrongly assumes that contributing laboratories have funding for this response and lack only the means to invest in long-term capacity building. The ultimate goal should be to have sufficient regional capacity and links to centres of expertise around the world. That avoids the need for “flying laboratories” and, of course, this requires reliable and long-term funding for the whole network. Some government and other health funders have realized this need and have recently set up a new funding mechanism to develop vaccines for priority emerging infectious diseases: the Coalition for Epidemic Preparedness Innovations (CEPI). We need something similar for generic preparedness.

Q: During the SARS outbreak in 2003, scientists agreed to share their findings, in particularly the genetic sequences of the pathogen, leading to its identification. Is this a model that should be widely replicated?

A: The sharing of data for SARS was great and allowed scientists to identify the pathogen causing SARS. Yet in reality this data sharing was limited to a small group of scientists and WHO, until their findings were published. At that time I was heading the emerging disease laboratory response in the Netherlands and we did not have access to the information we needed to do diagnostic tests for SARS, for example, for travellers. Research and the public health response need to be more closely linked as, for example, the EVD-LabNet in Europe for emerging viruses and laboratory preparedness, in which data is shared promptly. There are similar regional networks in other parts of the world.

Q: How can scientists be incentivized to act in the interests of public health?

A: Many scientists are interested in public health, but may not be familiar with how the public health sector works. The question of incentives is more complex than public health versus academia. There is no single solution, but several potential solutions. These include data sharing platforms that work within a code of conduct and that facilitate the sharing and use of data among a range of stakeholders for public health action, while retaining the right to publication or set out rules for use by the private sector. An example of such rules is that sharing needs to be immediate in the case of a public health emergency. We are working on such a platform through a collaborative group in Europe called Compare.

Q: Indonesia refused to share data on A (H5N1) influenza virus arguing that its population would not benefit from new, expensive vaccines produced using their data. What is needed to incentivize countries to share their data with the international community to come up with shared solutions to emerging pathogens?

A: Withholding data is not the way forward, but the Indonesians had a point regarding affordability. On the other hand, we need private sector expertise to bring vaccines, diagnostics and therapeutics to the market, if their economic interests are not protected, they may not be interested. So a middle ground needs to be found. The current emphasis on access and benefit sharing, as envisaged by the *Nagoya Protocol on Access to Genetic Resources* is a step forward. This agreement makes national authorities, not individual scientists or institutions, responsible for deciding what is shared and what is not shared. But not all countries are signed up to it and the Nagoya protocol was not developed with infectious diseases in mind.

“Global health security is as weak as the weakest links in the chain.”

Q: You have been involved in the WHO R&D Blueprint project, can you tell what this is?

A: The R&D Blueprint project set out to evaluate what went wrong with recent outbreak responses, and how these should be done in future and to push for concrete action. The project’s priority list of about 10 emerging pathogens and the CEPI initiative to start developing vaccines for some priority diseases are good examples of such action. However, it is important to keep up the momentum, particularly for the practical side of preparedness and response. Global health security is as weak as the weakest links in the chain, so how do we build global disease preparedness and response capacity that combines diagnostic preparedness with the ability to support outbreak research? Currently, the development of laboratory capacity is a local or national responsibility. So here, again, the plea for a global, publicly-funded network of emerging infectious disease preparedness and response hubs. Such a project needs dedicated staff, equipment, facilities, data-sharing systems prepared during “peace time”, so that they can be deployed during outbreaks. Given that many new diseases arise from our interaction with animals, a significant part of this network should be dedicated to regionally dispersed One Health centres of excellence. This project may seem expensive, but is not when compared to the economic costs of recent major outbreaks.

Q: The 2014–16 West African Ebola outbreak, which was not recognized for months after the first cases, incurred huge economic costs. How can least-developed countries with a weak infrastructure be expected to do the surveillance needed to detect outbreaks? Is implementation of the International Health Regulations (IHR) the solution?

A: IHR implementation is part of the solution, but preparedness and response will always be a low priority when there are so many more diseases to deal with every day. This dilemma is not limited to low-income settings. Clinicians and public health institutes across the world face the challenge of working with limited budgets, shifting the emphasis to chronic diseases (often from the same budget), and the growing costs of health care due to the ageing population. WHO relies on many partners for the outbreak response, but does not have a mechanism to fund those response activities. That situation may have been acceptable in the past, but not for today’s emerging infectious diseases which require a more consistent approach.

Q: WHO was accused of scaremongering during the A (H1N1) 2009 influenza pandemic, as the illness turned out to be mild and case fatality was lower than expected. During the 2014–16 Ebola outbreak, WHO was accused of failing to pay enough attention. How do you decide which emerging infectious pathogens and outbreaks present a major risk for humans and merit action, without scaremongering?

A: It’s easy to judge events with hindsight. In 2009, the initial reports from Mexico were of great concern and it was difficult to assess the risk because there is no globally agreed way of assessing severity during early stages of an outbreak unless you do a series of studies. Fortunately, the pandemic turned out to be milder than all the worst-case scenarios. But while a situation is evolving, you still have to decide on vaccine development for all scenarios. This was a global pandemic, so that assessment was right, although the severity prediction was wrong.

Q: What is your view on the Global Virome Project to identify all potentially new and emerging viruses in the natural world?

A: This is an interesting research project, but I am sceptical about the claims made by some scientists that all emerging viruses can eventually be discovered. Virome studies are increasingly popular and reveal that we are far from understanding the full spectrum of viral diversity. That said, I do believe in the power of metagenomic sequencing and other new technologies, preferably when these activities are integrated into the network that I would like to see develop.

